# Epigenetics of Cutaneous Sarcoma

**DOI:** 10.3390/ijms23010422

**Published:** 2021-12-31

**Authors:** Emi Mashima, Yu Sawada

**Affiliations:** Department of Dermatology, University of Occupational and Environmental Health, 1-1, Iseigaoka, Yahatanishi-Ku, Kitakyushu 807-8555, Japan; e-mashima@med.uoeh-u.ac.jp

**Keywords:** skin sarcoma, epigenetics, dermatofibrosarcoma protuberans, angiosarcoma, Kaposi’s sarcoma, leiomyosarcoma, liposarcoma

## Abstract

Epigenetic changes influence various physiological and pathological conditions in the human body. Recent advances in epigenetic studies of the skin have led to an appreciation of the importance of epigenetic modifications in skin diseases. Cutaneous sarcomas are intractable skin cancers, and there are no curative therapeutic options for the advanced forms of cutaneous sarcomas. In this review, we discuss the detailed molecular effects of epigenetic modifications on skin sarcomas, such as dermatofibrosarcoma protuberans, angiosarcoma, Kaposi’s sarcoma, leiomyosarcoma, and liposarcoma. We also discuss the application of epigenetic-targeted therapy for skin sarcomas.

## 1. Introduction

### 1.1. The Skin as an Exposure Site to Environmental Stimuli

Various environmental stimuli have their most direct effect on the skin, the outermost organ that covers the human body (reviewed in [1]). The skin is the most susceptible organ to various external factors, and continuous skin reactions to environmental factors augment tissue damage caused by inflammatory reactions [2]. In recent studies, skin physiology has been found to involve many epigenetic mechanisms that regulate gene expression without changing genetic information [3] (reviewed in [4,5]). In addition to playing a role in adaptation to environmental conditions, epigenetics also contributes to carcinogenesis in skin cancers (reviewed in [6,7]). Thus, it has been hypothesized that cutaneous malignancies may acquire a selective advantage within the body via an epigenetic mechanism.

### 1.2. The Skin Structure and Functions

The skin is a three-layered structure consisting of the epidermis, dermis, and adipose tissue, each with unique cells, and its importance has been investigated in various studies [8,9]. It plays essential roles in maintaining homeostasis in the human body, such as having barrier, sensory, thermoregulatory, immune, and secretory functions (reviewed in [10]). Keratinocytes produce moisturizing factors to increase the water retention capacity of the stratum corneum, thus contributing to skin barrier function [11]. Keratinocytes, dendritic cells, and lymphocytes in the skin play a vital role in immunity [12]. These cells carry Toll-like receptors, which recognize the specific molecular pattern of invading pathogens and activate innate immunity to promote the production of various cytokines and antimicrobial peptides. Skin also plays an important role as a sensory organ (reviewed in [13]). Itch exists only in the skin, and the scratching action that accompanies itching is greatly involved in the exacerbation of skin diseases [14]. In addition, proper thermoregulation of the skin surface, which involves the dilation of capillary vessels and sweating, is essential for maintaining homeostasis. Hair follicles, sebaceous glands, and sweat glands are mini-appendages that produce sebum and sweat, which can lead to skin diseases (reviewed in [15]). Various types of soft tissue tumors are known to develop in the skin. As the skin is susceptible to epigenetic modification by environmental factors, it has been inferred that these skin-derived, soft-tissue tumors might be affected by these mechanisms.

### 1.3. Epigenetic Changes in the Skin

Epigenetic chemical modifications of deoxyribonucleic acid (DNA) or DNA-binding histones regulate gene transcription without changing the DNA sequence (reviewed in [16]). DNA binds histones via electrostatic interactions. DNA transcription is affected by histone binding; for example, transcription is activated when histone–DNA bonds are disrupted, which is considered an epigenetic mechanism. Environmental stimuli have been hypothesized to promote oncogenesis in the skin via epigenetic mechanisms. Therefore, elucidating the detailed molecular mechanism of epigenetic changes in cutaneous malignancies may be critical for our understanding of skin cancers.

In this review, we focus on soft-tissue sarcomas affected by epigenetic regulation mechanisms and clarify the relationship between their initiation and epigenetics. Furthermore, we discuss the future direction of epigenetic-targeted therapy for cutaneous soft-tissue sarcomas. We highlight the molecular mechanisms of epigenetic modification in cutaneous sarcomas, such as dermatofibrosarcoma protuberans (DFSP), angiosarcoma, Kaposi’s sarcoma, leiomyosarcoma, and liposarcoma.

## 2. Dermatofibrosarcoma Protuberans (DFSP)

DFSP is a rare cutaneous sarcoma derived from fibroblasts and is mainly located in the dermis [17]. It has an indolent clinical course. Although surgical resection is the gold standard treatment, there is no effective therapeutic option to prevent local aggressive invasion (although distant metastasis rarely occurs) [18]. Therefore, various studies are currently exploring therapeutic candidates for DFSP. COL1A1–PDGFB fusion has been identified in DFSP cases [19]. COL1A1–PDGFB fusion variants frequently involve exons 25, 32, and 47, suggesting possible pathogenesis of DFSP [19]. The incidence of DFSP is 4.2 cases per one million persons per year [20]. It accounts for 4.5% of all cutaneous soft tissue sarcomas [21] and approximately 1.8% of all soft tissue sarcomas (reviewed in [22]).

A possible contribution of histone methylation to DFSP has been previously reported. One of the inducers of histone methylation, polycomb repressive complex 2 (PRC2), primarily tri-methylates histone H3 on lysine 27 to form H3K27me3, and it is required for the initial silencing of targeted genes [23]. The core complex of PRC2, consisting of enhancer of zest homolog (EZH) 1 and EZH2, the zinc-finger protein SUZ12, and the WD40 protein EED [24,25,26,27,28], is necessary for its catalytic function. EZH2 is the major catalytic subunit of PRC2 and the histone methyltransferase that catalyzes H3K27 trimethylation (reviewed in [29]). Whole-transcriptome sequencing analysis showed a strong upregulation of EZH2 in DFSP, possibly associated with the epithelial–mesenchymal transition [30]. EZH2 enhances cell proliferation and is widely found in cancers, including renal cancer [31], breast cancer [32], prostate cancer [33], and lymphoma [34]. Therefore, EZH2-mediated histone methylation might cause transcriptional repression of key tumor suppressor genes, such as p53 [35], and contribute to the development of DFSP.

## 3. Angiosarcoma

Angiosarcoma is a malignancy of endothelial cells in the blood or lymphatic vessels (reviewed in [36]). High frequencies of local recurrence and distant organ metastases have been reported. Current therapeutic options for high-grade malignancies are not satisfactory. The incidence of angiosarcoma is 1.5 cases per one million persons per year (reviewed in [37]). It accounts for 0.4% of all cutaneous soft tissue sarcomas [21], and approximately 2.0% of all soft tissue sarcomas [38].

**Figure 1:**
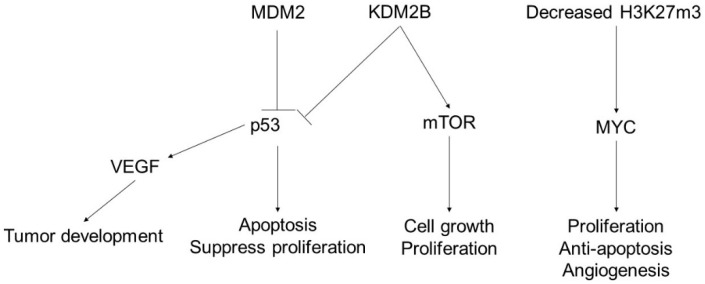
The pathogenesis of angiosarcoma and the influence of epigenetics.

The oncogenesis of angiosarcoma is shown in Figure 1. The p53/mouse double minute 2 homolog (MDM2) pathway has been postulated to be central to the development of angiosarcoma [39,40]. MDM2 suppresses p53 activity and vascular endothelial growth factor (VEGF) [39]. Intact p53 acts as a negative driver of the VEGF gene, thus inhibiting angiogenesis [39]. The suppression of p53 by MDM2 enhances angiogenesis in angiosarcoma cells, suggesting that p53 suppresses MDM2-induced angiosarcoma growth [41]. A high incidence of angiosarcoma has been observed in p53-deficient mice [42]. MYC is a multifunctional nuclear protein that is related to the cell cycle, apoptosis, angiogenesis, and metastasis of angiosarcoma (reviewed in [43]). MYC upregulation was observed in secondary angiosarcoma and to a lesser extent in primary angiosarcoma. The mammalian target of the rapamycin (mTOR) pathway is involved in angiosarcoma onset [44]. mTOR enhances cancer cell growth and proliferation, and mTOR inhibitors inhibit growth in a dose-dependent manner [45].

Histone demethylation is involved in the development of angiosarcoma. H3K27me3 deficiency is observed in radiation-associated angiosarcoma [46]. As histone methylation causes downregulation of targeted genes [47,48], it has been speculated that epigenetic modulators are involved in histone methylation. Lysine-specific demethylase 2b (KDM2B) is a Jumonji domain histone demethylase [49] and is a component of PRC1 [50]. PRC1 is part of a polycomb repressive complex that inhibits the activated form of the RNA polymerase II preinitiation complex assembly by using immobilized H3K27-methylated chromatin [51]. KDM2B regulates various physiological and pathological processes, such as proliferation and oncogenesis [52]. KDM2B is upregulated in angiosarcomas [53], where it suppresses p53 and activates mTOR signaling (reviewed in [54]). KDM2B silencing in angiosarcoma cells enhances cell death via inactivation of DNA repair. The histone demethylase inhibitor GSK-14 induces apoptosis and cell death and reduces tumor size [53]. MYC upregulation is closely associated with radiation-related angiosarcoma due to H3K27me3 deficiency [46].

In angiosarcoma, p53 is a tumor suppressor that enhances apoptosis and suppresses tumor cell proliferation. The impairment of p53 function enhances VEGF function and causes tumor development. In addition, mTOR and MYC positively drive cell growth and angiogenesis. In these tumorigenesis mechanisms, KDM2B is upregulated in angiosarcoma, suppresses p53, and activates mTOR signaling. This leads to the suppression of apoptosis and enhancement of cell growth and proliferation. MYC upregulation is closely associated with radiation-related angiosarcoma due to H3K27me3 deficiency.

## 4. Kaposi’s Sarcoma

Human herpesvirus-8, also known as Kaposi’s sarcoma-associated herpesvirus (KSHV), is an opportunistic pathogen infecting vascular endothelial cells in immunocompromised people, such as those on immunosuppressive agents (e.g., after organ transplantation) and AIDS patients (reviewed in [55]). KSHV is a double-stranded DNA virus that causes either latent or lytic infections and infects a range of cells, including endothelial and immune cells. Patients with Kaposi’s sarcoma develop purpura or dark brown macules or plaques, which are prone to bleeding and ulceration. Kaposi’s sarcoma in patients with untreated acquired immunodeficiency syndrome (AIDS) can spread to the rest of the body within a few months. Locally limited Kaposi’s sarcoma of the skin can be removed by surgical resection [56]. However, patients who must continue to receive immunosuppressive drugs, chemotherapy, and radiation therapy are particularly susceptible to progression to advanced Kaposi’s sarcoma. The incidence of Kaposi’s sarcoma is approximately 0.2 cases per 100,000 persons per year [57]. It accounts for 17.1% of all cutaneous soft tissue sarcomas [21], and approximately 3.3% of all soft tissue sarcomas [57].

**Figure 2:**
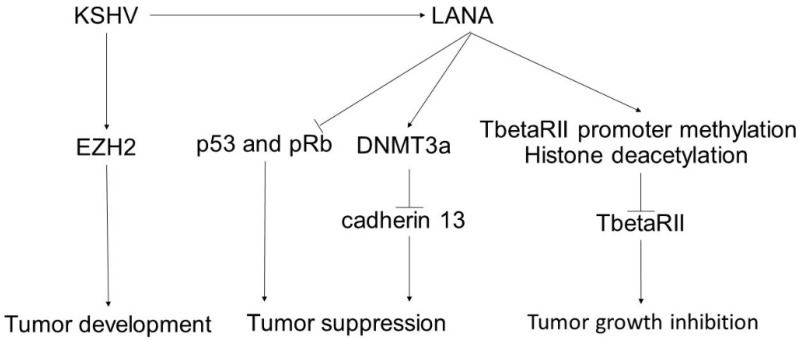
The pathogenesis of Kaposi’s sarcoma and the influence of epigenetics.

KSHV-associated LANA inactivates p53 and pRb [58], which blocks cell cycle arrest and causes uncontrolled proliferation [59] (Figure 2). LANA also binds to glycogen synthase kinase 3b (GSK-3b) [60,61], which increases the level of β-catenin (reviewed in [62]), facilitating tumor progression through the induction of angiogenesis [63]. LANA acts via methylation of the TbetaRII promoter and deacetylation of histones, which leads to inhibition of TGF-β signaling [64]. LANA also cooperates with DNA methyltransferase 3a (DNMT3a), which is a representative driver for DNA methylation in CpG-enriched DNA sites, especially targeted gene promoter regions (reviewed in [65]) and facilitates promoter methylation of cadherin 13 [66]. Cadherin 13 is a member of the cadherin family that is often downregulated in various tumors and is associated with an unfavorable prognosis [67]. Cadherin 13 inhibits cell proliferation and tumor invasion and increases apoptosis (reviewed in [68]). Therefore, LANA-mediated cadherin 13 downregulation contributes to the development of Kaposi’s sarcoma.

KSHV also encodes viral interleukin-6, which contributes to the development of Kaposi’s sarcoma [69,70]. Viral interleukin-6 promotes cell proliferation, tumor invasion, and angiogenesis by suppressing caveolin 1, which plays a role in tumor development mediated by DNA methyltransferase 1 (DNMT1)-targeted STAT3 acetylation [69].

EZH2 upregulation is related to epigenetic changes in KSHV and contributes to the development of KSHV-induced oncogenesis and angiogenesis [71].

KSHV-associated LANA inactivates p53 and pRb, enhances cell cycle arrest, and causes tumor cell proliferation. LANA acts via methylation of the TbetaRII promoter and deacetylation of histones, which leads to the inhibition of TGF-β signaling. LANA cooperates with DNMT3a and facilitates promoter methylation of cadherin 13 to cause its downregulation in various tumors, leading to cell proliferation, tumor invasion, and suppression of apoptosis. Therefore, LANA-mediated cadherin 13 downregulation contributes to the development of Kaposi’s sarcoma. KSHV promotes tumor development mediated by EZH2 activation.

## 5. Leiomyosarcoma

Leiomyosarcoma is a form of malignant smooth muscle tumor and is one of the most common types of soft-tissue sarcomas (reviewed in [72]). Dermal leiomyosarcoma develops from the pilar follicle erector muscles or smooth muscles surrounding the sweat glands, and subcutaneous leiomyosarcoma develops from the smooth muscle of arteries and veins [73]. The incidence of leiomyosarcoma is between 0.7 cases per 100,000 persons per year [57]. It accounts for 0.6% of all cutaneous soft tissue sarcomas [21] and approximately 11.4% of all soft tissue sarcomas [57].

DNA methylation of DAXX and receptor-type tyrosine-protein phosphatase N2 (PTPRN2) has been found in patients with leiomyosarcoma [74]. Hypermethylation-mediated downregulation of soft tissue leiomyosarcoma-related genes, such as potassium voltage-gated channel subfamily A regulatory beta subunit 3 (KCNAB3), have been observed [75]. The actual role of KCNAB3 hypermethylation remains unclear, and a genome-wide analysis revealed hypermethylation in KCNAB3 in pre-cancer (reviewed in [76]), suggesting that this alteration is a positive driver for oncogenesis.

Hypermethylation in the potassium voltage-gated channel, Isk-related family, member 3 (KCNE3), TSPY-like 5 (TSPYL5), Homeobox A11 (HOXA11), HOXA11 antisense RNA (HOXA11AS), HOX9, and LOC100130872, was observed in leiomyosarcoma [75]. These genes are related to epigenetic regulation mediated by epigenetic modulators such as EZH2, histone 3 lysine methyltransferase 2A (KMT2A), BRD4, and histone deacetylase 4 (HDAC4) [75]. KMT2A has a SET domain that is responsible for its histone H3 lysine 4 (H3K4) methyltransferase activity in cancers (reviewed in [77,78]). BRD4 is an epigenetic regulator and has two tandem bromodomains (BD1 and BD2), which bind acetylated lysine residues on histones and regulate gene transcription (reviewed in [79]). Leiomyosarcoma growth is suppressed by treatment with DNA methyltransferase inhibitors [80].

HDAC9 can influence H3K27 acetylation in leiomyosarcoma and enhance cell survival by repressing Fas cell surface death receptor (FAS) transcription [81]. The addition of valproic acid (VPA), a weak histone deacetylase inhibitor, combined with gemcitabine and docetaxel, has cytotoxic effects [82]. Patients with extrauterine leiomyosarcomas showed a partial response after VPA treatment.

## 6. Liposarcoma

Liposarcoma is a malignant soft tissue tumor located in the deep soft tissues of the extremities [83]. Epigenetic effects have been reported in several types of liposarcomas, such as atypical lipomatous tumor (ALT)/well-differentiated liposarcoma, myxoid/round-cell liposarcoma (MRCL), and dedifferentiated liposarcoma. The incidence of liposarcoma is approximately 0.7 cases per 100,000 persons per year [57]. It accounts for 0.1% of all cutaneous soft tissue sarcomas [21] and less than 20% of all soft tissue sarcomas (reviewed in [84]).

Evans et al. reported that well-differentiated liposarcomas—namely, ALT, located in a subcutaneous site or within a muscle layer have high frequencies of local recurrence and distant metastasis [85]. ALT is associated with gene amplification in the 12q12-21 and 10p11-14 regions and of *MDM2* and cyclin-dependent kinase 4 (*CDK4*) [86]. Well-differentiated liposarcomas rarely metastasize and are often associated with p16INK4a deficiency. Gene silencing by methylation of the *p16INK4a* gene promoter has been reported in the progression and dedifferentiation of well-differentiated liposarcoma [87].

MRCL is a subtype of liposarcoma that usually occurs in the extremities and often spreads throughout the body (reviewed in [88]). Typical myxoid liposarcomas exhibit nonpleomorphic ovoid mesenchymal cells with a myxoid matrix. Round-cell liposarcoma is a high-grade form of round mesenchymal cell liposarcoma without a myxoid matrix. The most common cytogenetic feature of MRCL, in 95% of cases, is chromosomal translocation t(12;16)(q13;p11) producing FUS RNA-binding protein (FUS)–DNA damage-inducible transcript 3 (DDIT3) fusion proteins [89]. The remaining 5% cases of MRCL involved a t(12;22)(q13;q12) chromosomal translocation, producing EWS RNA-binding protein 1 (EWSR1)–DDIT3 fusion proteins [90]. These translocations can be used as diagnostic indicators (reviewed in [91]). The FUS–DDIT3 fusion gene enhances MRCL infiltration by inhibiting the expression of miR-486, which plays an inhibitory role in MRCL cell growth. Tumors with a methylated adenomatous polyposis coli (APC) promoter showed downregulation of APC, which acts as a scaffolding protein for the regulation of oncoprotein beta-catenin [92].

**Figure 3:**
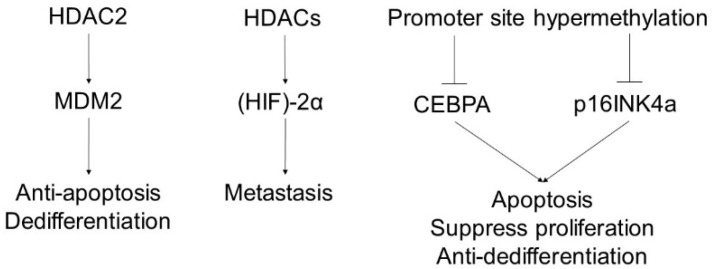
The pathogenesis of liposarcoma and the influence of epigenetics.

Many genetic mutations have been reported in dedifferentiated liposarcomas, including mutations in *MDM2, CDK4*, high mobility group AT-hook 2 (*HMGA2*), fibroblast growth factor receptor substrate 2 (*FRS2*), and neuron navigator 3 (*NAV3*), all located in 12q13-15 [93] (Figure 3). The p16INK4a gene promoter is also hypermethylated in 50% of dedifferentiated liposarcomas [94]. Somatic mutation of *HDAC1* was identified in 8.3% of patients with dedifferentiated liposarcoma [95]. HDAC2 was highly co-expressed with MDM2, and pharmacological HDAC2 inhibition decreased MDM2 expression and enhanced tumor cell apoptosis [96]. CCAAT/enhancer-binding protein alpha (CEBPA) methylation was observed in 24% of patients with dedifferentiated liposarcomas. Pharmacological demethylation restored CEBPA expression in DLPS cells, suppressing proliferation and enhancing apoptosis [95]. Hypoxia-inducible factor (HIF)-2α activates gene transcription under hypoxic conditions and promotes metastasis of soft-tissue sarcomas. The HDAC inhibitor vorinostat inhibits tumor growth mediated by HIF-2α [97]. H3K9me3 is enhanced in dedifferentiated liposarcoma [98].

MDM2 is responsible for the development of dedifferentiated liposarcomas that cause antiapoptotic effects and the dedifferentiation of tumors. HDAC2 is co-expressed with MDM2, and HDAC2 inhibition results in decreased MDM2 expression and enhanced tumor cell apoptosis. CEBPA and p16INK4a act as suppressors of liposarcoma to induce apoptosis and suppress proliferation and dedifferentiation. Hypermethylation of CEBPA and p16INK4a is observed in liposarcoma and causes downregulation and enhances tumor development. HIF-2α activates gene transcription under hypoxic conditions and promotes metastasis in liposarcomas. Therefore, HDAC inhibitors inhibit tumor growth mediated by HIF-2α.

## 7. Radiotherapy-Induced Epigenetics Alteration

Radiation therapy is a representative treatment for soft tissue sarcomas [99]. Recent studies have identified alterations in epigenetics following radiotherapy. Changes in DNA methylation indicated a potential epigenetic response to ionizing radiation in MDA-MB-231 cells, and methylated genes were enriched in gene ontology categories related to cell cycle, DNA repair, and apoptosis pathways [100]. These actions are expected to improve the therapeutic efficacy of radiotherapy against cutaneous sarcomas. Radiation also alters histone H3 methylation profiles [101]. The decreased expression of H3K27me3 is observed in almost all cases of radiation-related angiosarcoma, suggesting that histone methylation might contribute to the development of angiosarcoma following radiation [46]. This indicates that radiation might have bi-directional effects on the development and regulation of cutaneous sarcoma.

## 8. Summary of Epigenetic Modification in Cutaneous Sarcoma and Therapeutic Application

Finally, we summarize the roles of epigenetic targets and epigenetic-modifying enzymes in Table 1. Table 2 shows the possible therapeutic options for skin sarcomas. Various epigenetic enzyme inhibitors are currently being tested in clinical trials for the treatment of malignancies and inflammatory diseases. As there are several epigenetic-modifying enzymes involved in skin cancers, we discuss the clinical application of these inhibitors in cutaneous sarcomas.

EZH2 activation is involved in DFSP, MPNST, angiosarcoma, Kaposi’s sarcoma, leiomyosarcoma, epithelioid sarcoma, and synovial sarcoma. Tazemetostat is a selective, reversible, small-molecule inhibitor of EZH2 that has been used in clinical trials for refractory non-Hodgkin’s B-cell lymphoma [102,103,104], diffuse large B-cell lymphoma [105], follicular lymphoma [106], and advanced epithelioid sarcoma [107]. Due to the role of EZH2 in skin sarcomas, tazemetostat may be effective against these cancers.

DNA methyltransferases (DNMTs) are a family of enzymes that catalyze the transfer of methyl groups to DNA (reviewed in [108]) and play a role in a wide range of biological functions. DNMT1 and DNMT3B are involved in Kaposi’s sarcoma, and DNMTs are upregulated in leiomyosarcoma. MG98, which inhibits DNMT1 production, has antitumor effects in advanced renal cell carcinoma [109]. Another DNMT1 inhibitor, decitabine, has antitumor effects in patients with myelodysplastic syndrome [110]. SGI-1027 is another DNMT1 inhibitor that showed synergistic antitumor effects in combination with a JAK1/2 inhibitor [111].

Histones contain many basic amino acids, such as lysine and arginine, which are positively charged and can bind to negatively charged DNA (reviewed in [112]). Histone acetylation by histone acetyltransferase converts the amino group (-NH2) of a specific lysine residue in histone to amide (-NHCOCH3), neutralizing the charge (reviewed in [113]). Partial weakening of the bonds between DNA and histones makes it easier for transcription factors and RNA polymerases to bind to the DNA strand and activate gene transcription (reviewed in [114,115]). In contrast, histone deacetylation by HDAC has the opposite effect, causing the DNA to condense around histones, suppressing transcription (reviewed in [116]). HDAC4 and HDAC9 are upregulated in leiomyosarcoma, and HDAC2 is activated in liposarcomas. A variety of HDAC inhibitors that inhibit multiple HDACs are currently available. As pan-HDAC inhibitors show antitumor effects in cutaneous sarcoma, current specific HDAC inhibitors may be used against skin sarcomas. HDAC4 is upregulated in leiomyosarcoma, and LMK235, a specific HDAC4 inhibitor, dose-dependently suppressed MKK7 transcription and JNK/c-Jun activity in early brain injury following subarachnoid hemorrhage and impaired neuronal apoptosis [117]. HDAC2 is enhanced in liposarcoma, and Ohio State University HDAC42 (OSU-HDAC42) is a selective HDAC2 inhibitor that has been examined in colon cancer [118]. The HDAC2 inhibitor CAY10683 ameliorates LPS-induced inflammation in neurons [119] and acute liver dysfunction [120]. Pan-HDAC inhibitors are known to have therapeutic efficacy against cutaneous sarcomas. The HDAC inhibitors romidepsin and quisinostat can suppress tumor growth in synovial sarcoma [121,122]. Vorinostat is currently available in clinics [123]. Other HDAC inhibitors are also expected to be useful in treating cutaneous sarcomas.

Current advancements in immunotherapy against malignancies have improved the clinical outcomes for the treatment of advanced or metastasis-form cancers. Sarcomas are also expected to be treatable using immunotherapy. Specifically, PD-1-targeted therapy has been highlighted in sarcomas [124]. Class I HDAC is associated with an unfavorable prognosis in patients with sarcomas, and its inhibition by chidamide, in combination with anti-PD-1 antibody treatment, improved survival in mice [125].

Although these epigenetic-targeted treatments have been explored to a modest degree for cutaneous sarcoma, further clinical trials are needed to clarify their impact and future utility.

## 9. Conclusions

Herein, we reported the effects of epigenetic modifications on skin soft-tissue malignancies and ways to target such modifications for future therapeutic applications. Epigenetics comprehensively affects soft tissue malignant tumors. As oncogenesis depends on the characteristics of each originating cell type, the influence of specific epigenetic modifications must be studied in each tumor type. Based on the promising antitumor effects of epigenetics-targeted therapy on cutaneous sarcomas in previous studies, clinical studies are needed to further explore this option. As curative treatment for soft-tissue sarcomas is still difficult to achieve, the development of epigenetic-targeted treatment for this tumor type could hold great potential.

## Figures and Tables

**Table 1:** Epigenetic modifications in skin cancers.

Cutaneous Sarcomas	Epigenetic Modulator	Histone Modification	DNA Modification
DFSP	EZH2↑ [30]		
Angiosarcoma	KDM2B↑ [53]	H3K27me3↓ [46]	
Kaposi’s sarcoma	DNMT1↑ [69] DNMT3A↑ [66] EZH2↑ [71]		Methylation TbetaRII↑ [64]
Leiomyosarcoma	EZH2↑ [75]. KMT2A-AFDN↑ [75] BRD4↑ [75] HDAC4↑ [75] HDAC9↑ [81] DNMT↑ [80]		Methylation DAXX↑ [74] PTPRN2↑ [74]
Liposarcoma	miR-486↑ [92] HDAC2↑ [96]	H3K9me3↑ [98]	Methylation p16INK4a↑ [94] APC↑ [92] C/EBPα↑ [95].

**Table 2:** The potent therapeutic efficacy against skin sarcomas.

Candidates	Action Mechanism
Tazemetostat	EZH2 inhibitor [102,103,104,105,106,107]
MG98	DNMT1 inhibitor [109]
Decitabine	DNMT1 inhibitor [110]
SGI-1027	DNMT1 inhibitor [111]
Quisinostat and romidepsin	Pan-HDAC inhibitor [121,122]
OSU-HDAC42	HDAC2 inhibitor [118]
CAY10683	HDAC2 inhibitor [120]
LMK235	HDAC4 inhibitor [117]

## Data Availability

Not applicable.
